# Analysis and Reduction of Solar Stray Light in the Nighttime Imaging Camera of Luojia-1 Satellite

**DOI:** 10.3390/s19051130

**Published:** 2019-03-06

**Authors:** Xing Zhong, Zhiqiang Su, Guo Zhang, Zhigang Chen, Yao Meng, Deren Li, Yong Liu

**Affiliations:** 1 Chang Guang Satellite Technology Co., Ltd., Changchun 130102, China; suzhiqiang927@163.com (Z.S.); asir0422@163.com (Z.C.); mengyaosatellite@hotmail.com (Y.M.); liuyongafighter@163.com (Y.L.); 2 Changchun Institute of Optics, Fine Mechanics and Physics, Chinese Academy of Sciences, Changchun 130033, China; 3 School of Remote Sensing and Information Engineering, Wuhan University, Wuhan 430079, China; guozhang@whu.edu.cn; 4 State Key Laboratory of Information Engineering in Surveying, Mapping and Remote Sensing, Wuhan University, Wuhan 430079, China; drli@whu.edu.cn

**Keywords:** Luojia-1 satellite, nighttime imaging camera, stray light

## Abstract

As one of the experimental payloads on Luojia-1 satellite, the nighttime imaging camera works with a high sensitivity to acquire nighttime light on earth. Solar stray light is a fatal problem for optical satellite works in the polar orbit, even for nighttime scene imaging, resulting in image saturation and light signal detection failure. To solve this problem, an analysis of the range of solar incident angles was conducted firstly. Based on the result, a special-shaped baffle was designed to avoid direct sunlight incidence. Moreover, the capability of stray light elimination of the lens was enhanced by an order of magnitude via optimizing the internal structure. An evaluation of secondary scattering stray lights into the camera from surrounding parts was performed based on a real satellite model. The results showed that the stray light elimination reaches a 10^−10^ order, meeting design requirements. Utilizing on-orbit images, the ability of satellites in illuminated areas to obtain artificial lights in dawn-dusk area was verified, proving the effectiveness of the stray light elimination design.

## 1. Introduction

Nighttime remote sensing is the process of using optical sensors to obtain images of the earth’s surface during the nighttime [1,2]. The Operational Line Scan System (OLS) of the US Defense Meteorological Satellite Program (DMSP) was started in 1970s and was initially designed to capture the dim lunar light reflected by nighttime clouds, in order to obtain the distribution of clouds at night. It turned out that researchers found that DMSP/OLS could capture urban lights during cloudless nights. This is the origin of nighttime remote sensing [3,4,5,6]. Since then, many observation sensors, such as DMSP/OLS and VIIRS that can acquire nighttime visible and NIR images, were developed [7,8,9]. Nighttime images include not only urban lights but also lights from fishing vessels, natural gas combustion, forest fires, etc. [10]. These images are widely utilized in research areas, such as economy assessment, regional development research, major event evaluation, and fishery monitoring [11,12,13,14].

Luojia-1 satellite is a scientific experimental microsatellite jointly developed by Wuhan University and CGSTL (Chang Guang Satellite Technology Co., Ltd, Changchun, China). One of the experimental goals of Luojia-1 is to obtain nighttime remote sensing images for research in social macroeconomics. The ground sample distance (GSD) of the nighttime camera is 130 m while the swath width reaches 266 km. The Luojia-1 was launched on 2 June 2018 and has successfully acquired a lot of nighttime images, some of which have been put into preliminary applications [15,16,17,18].

The light source of daytime imaging is the sun, while nighttime imaging mainly relies on artificial lights. This results in special optical characteristics of nighttime observing targets, which are mostly ground with extremely low illumination or with illuminated urban areas with a big range of illuminance varies from several to tens of thousands lux [19]. It requires a high sensitivity and large dynamic range of the nighttime remote sensor.

**Figure 1:**
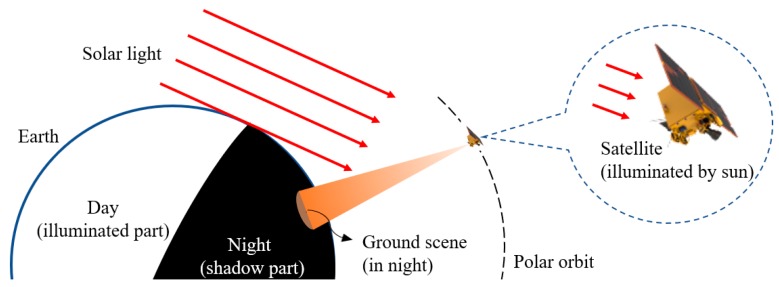
Solar light affection on nighttime image.

Stray light is one of the major aspects impacting the performance of optical sensors [20,21,22]. For sensors that work at daytime, normally stray light affects their average radiation level and quantification precision but not the ability to capture target images [23,24]. While for sensors working at nighttime, it needs more detailed analysis and assessment on the affection from stray light. The DMSP produces a stable product of a broad spectral band (0.4–1.1 μm) by filtering observations to remove the effects of moonlight, stray light, clouds, and ephemeral light sources [25]. For example, when analyzing the nighttime light images in the United States, only data from October to March covering the area from 20 to 55 degrees north latitude is selected due to solar stray light, otherwise stray light will present in the northern portions of images taken around summer solstice [26]. As for VIIRS, the specific analysis on stray light suppression was conducted during the design progress. VIIRS uses a Rotating Telescope Assembly (RTA) rather than a scan-mirror to perform cross-track scanning. Because the Primary Mirror in RTA is assembled deeper inside the scanning cavity, it thus prevents direct solar light incidence on the Primary Mirror. The instruments also contain a bulkhead to stop stray light passing from the RTA cavity into the aft optics afterwards. During normal VIIRS orbit, the solar vector is never within 28 degrees off the RTA optical axis, and the housing and baffles could completely block rays whose incident angle is more than 28 degrees. Thus, direct solar stray light is eliminated. However, there is still a stray light problem when imaging at high latitude areas, and it needs to be corrected using radiometry calibration data [27]. If the stray light is not properly eliminated, it may result in a false response or noise flooding the real light signal due to the low illuminance and large dynamic range of targets [28], especially when the satellite is capturing nighttime images of earth’s shadowed area, but with itself remaining in the solar illuminated area. This situation will occur when the satellite is imaging high-latitude areas, and then solar light becomes a major source of stray light, as shown in Figure 1.

As a microsatellite using refractive optical camera, the reduction of solar stray light is more difficult because of the camera’s envelop size limitation. During the development of nighttime camera on the Luojia-1, we performed an analysis based on the principle of internal and external stray light, and a solar vector model was established. The corresponding stray light elimination method including the special-shaped baffle and internal structure optimization was proposed. Moreover, an evaluation was performed with real illumination conditions, considering light scattered by surrounding components’ surfaces near the camera’s entrance, and the results showed the stray light was properly eliminated. After the satellite was successfully launched, in-orbit tests on stray light was conducted by analyzing the image obtained. The in-orbit test images (given in Section 6) shows that the solar light with incident angle more than 52 degrees was mostly barricaded and the rest was less than 10^−10^ order. 

## 2. Requirements on Nighttime Camera Stray Light

**Figure 2:**
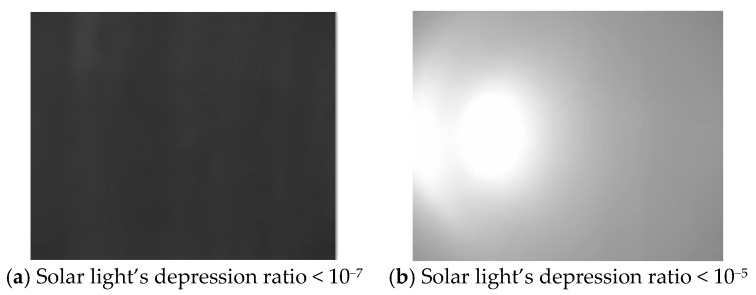
Typical images of stray light influences, using a collimated solar simulator and an imaging system with sensitivity of 10^−7^ solar flux.

Stray light in an imaging system is the ray that does not come from the target but reaches the sensor, or the one that comes from the target ending on the sensor via abnormal path. It confuses the energy distribution or even makes the detector totally saturated, then the dynamic range and clarity of the image will be reduced [29,30]. Figure 2 shows two typical images acquired by laboratory experiments, which use a collimated sun simulator to test the stray light response of some imaging system with sensitivity of 10^−7^ solar flux. It shows that, if the depressed solar light is much larger than the system’s sensitivity, the solar stray light has made serious effects on the image, and certainly cause the failure of a useful signal’s detection.

**Figure 3:**
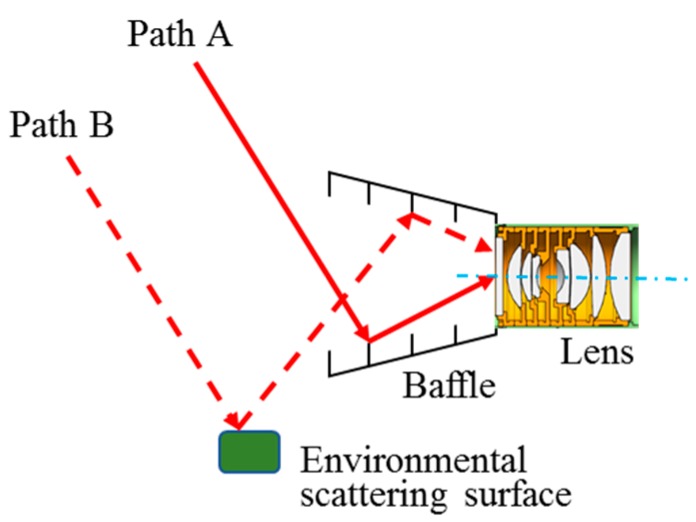
Stray light path.

The stray light in a nighttime camera mostly comes from external sources like the sun, moon, and atmospheric scattering. These rays enter the camera entrance via different paths and then reflect many times onto the sensor and cause distraction. For microsatellites like Luojia-1, their surfaces are covered by various components. Thus, the camera is threatened not only by rays through Path A, which go directly in the baffle, but also by those that travel through Path B and are scattered on environmental surfaces as shown in Figure 3. Both types of stray light need to be considered during the design.

Sunlight is the stray light source with the highest illuminance, it is reasonable to evaluate the stray light requirement in nighttime cameras by analyzing the contrast between stray sunlight signals and target signals. According to the design requirement, the estimated signal to noise ratio (SNR) of the Luojia-1 camera is above 20 dB for 10 lux ground illumination, with a dynamic range of 12 bit, while the noise-equivalent detecting illumination is 1 lux. Therefore, the illumination on the sensor caused by stray light should not affect the sensor acquiring signal of ground target of 1 lux.

Supposing a Lambertian target of reflectivity ρ and irradiation *E_G_*, the irradiation on the sensor pixel after traveling through atmosphere and the camera lens is [31]:

$$E_{t}=\frac{\rho \cdot E_{G}\cdot \tau_{0}\cdot \tau_{a}}{4F^{2}K_{\lambda }}$$

where $\tau_{0}$ is the total transmittance of about 0.8, $\tau_{a}$ the atmospheric transmittance of 0.6 according to the wavelength band calculation, F the F-number of camera lens which is 2.8 for Luojia-1. $K_{\lambda }$ is the conversion factor between illuminous flux and power, it equals 683 lm/W for 550 nm wavelength [32]. Thus $E_{t}=6.72\times 10^{-6}\;W/m^{2}$ for targets of 1 lux and ρ=0.3.

The lens performance of eliminating stray light is commonly evaluated by Point Source Transmittance (PST), which is defined as the ratio between the irradiance caused by point source of an off-axis angle θ on the sensor, and that on the aperture perpendicular to the point source [33,34]. Therefore, the irradiance on sensor caused by incident solar lights can be expressed as:

$$E_{S}=E_{sun}PST\left(\theta \right)$$

where $E_{sun}$ is the incident solar irradiance and θ the solar light incident angle off the optical axis.

According to the radiometric quality requirement, the energy of stray light should be less than 3% of the imaging energy, thus:

$$E_{S}<0.03E_{t}$$

Calculated by the solar spectral irradiance model, it is obtained that within the 500 nm~900 nm waveband where Luojia-1 camera works, the solar irradiance outside atmosphere is 608.6 W/m^2^, thus PST of this nighttime camera need to meet:

$$PST\left(\theta \right)<3.31\times 10^{-10}$$

Generally, a refractive lens could only reach a PST of 10^−5^ order itself, so an external baffle should be added. There are two types of external baffle—single-stage and multi-stage baffle. Stray light with certain off-axis angles could be eliminated by utilizing multi-stage external baffle whereas the baffle size could be huge, making it too difficult to apply on microsatellites [17]. Single-stage baffle is much smaller, and it can reduce the PST by two orders of magnitude, reaching 10^−7^ order, but apparently cannot meet the requirement of 10^−10^ for Luojia-1 nighttime camera. Therefore, a special-shaped baffle is proposed in this paper, according to the actual sunlight incident directions, aiming at keeping sunlight (Path A in Figure 3) from direct incident into the baffle. Moreover, as for the Path B situation, the inner structure of the camera lens is optimized in order to enhance the scattered stray light removing ability, and the environmental satellite components of the onboard camera are analyzed, using real satellite model, to evaluate the residual level of stray light.

## 3. Design of Special-Shaped Baffle

### 3.1. Analysis of Real Solar Incidence Angle

**Figure 4:**
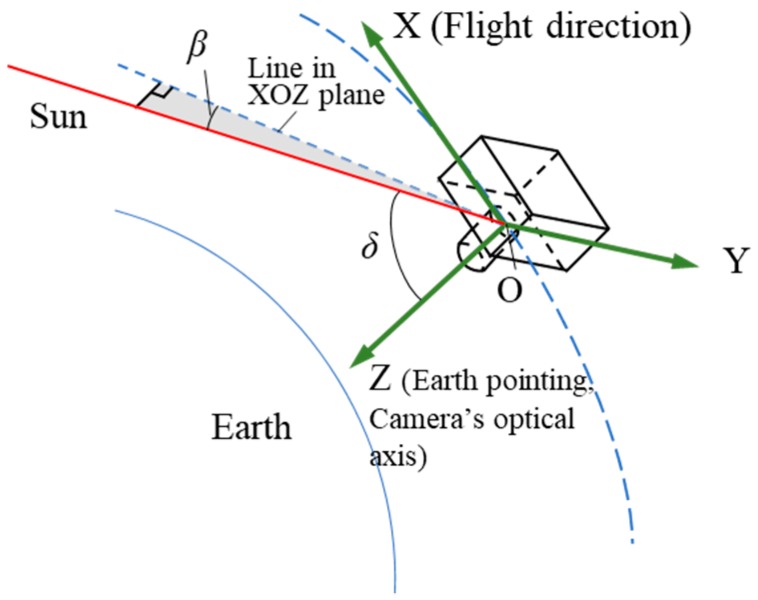
Layout of solar vector.

The Luojia-1 satellite works in a 650 km height orbit with the local time of the ascending node equator crossing as 22:40. It captures images of nighttime areas when traveling from south to north, as shown in Figure 4. This results in that the camera will get illuminated when performing tasks in high latitude area. The VS087 theory [18] provides a method to calculate the solar position in Earth Centered Inertial, which can be used to compute the incidence parameters of sunlight. By changing the solar coordinate, the solar position can be transformed to the orbit and satellite coordinate system, obtaining the incidence parameters of sunlight in reference to the orbit plane and satellite body.

**Figure 5:**
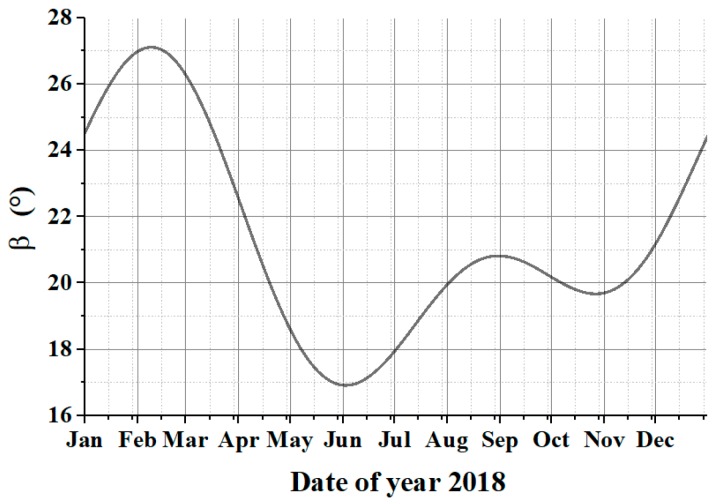
Angular change between solar vector and the orbit plane within a year.

According to the Luojia-1 orbit parameters, the variation of angle β between the solar vector and the orbit plane within a year can be calculated, using VS087 theory. The result is shown in Figure 5 (typically, for the sun-synchronous orbit, the curve is not purely sinusoidal). Under current orbit characteristics, β changes in years and normally stays in the −*Y* side of the orbit plane. The angular change is within 17°–27° while the average value is 22°.

Concluded from Figure 5, angle β reaches a minimum value on 3 June 2018. Sunlight distracts imaging mostly at that day. Thus, we could analyze the smallest angle between the solar vector and the camera’s optical axis when the satellite moves in the illuminated area, by considering the relative positions of the satellite, sun, and earth at 3 June 2018, as critical situation.

**Figure 6:**
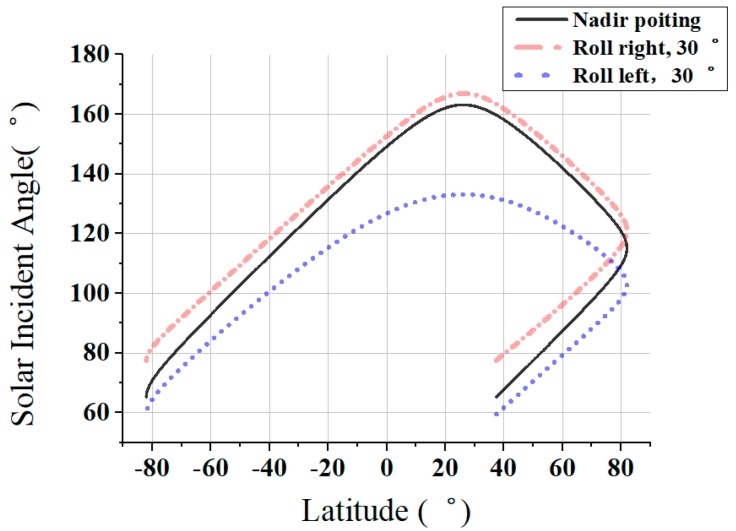
Angular change between solar vector and camera axis in one orbit cycle.

The attitude control system of Luojia-1 is mainly comprised by one digital sun sensor, two micro star trackers, one 3-axis magnetometer, three magnetic torquer rods and four flywheels (3 orthogonal, 1 diagonal), working in an earth-oriented three-axis stabilized attitude, while the yaw angle is automatically compensated during the imaging. The solar incidence angle β can be calculated via coordinate transform method [35]. Luojia-1 satellite is capable of swing up to ±30° (across-track only) according to the satellite overall requirements. Hence, the change of angle δ between the solar vector and camera optical axis in the illuminated area during one orbit cycle in 3 June 2018 was calculated under three different attitudes, as shown in Figure 6. The minimum value of δ is 52° when imaging in northern hemisphere.

### 3.2. Special-Shaped Baffle Design

**Figure 7:**
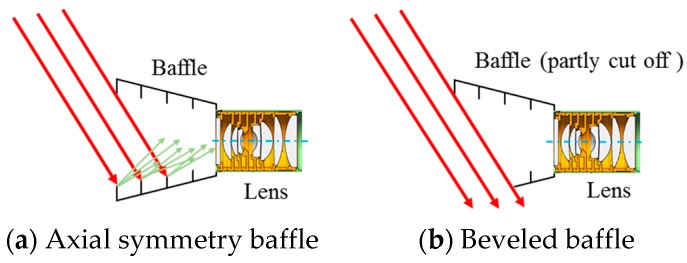
Principle of special-shaped baffle.

The special-shaped baffle is used to avoid direct incident stray light into the baffle. The principle is shown in Figure 7. Figure 7a shows the axial symmetry situation when the stray light is scattered by an internal structure and finally reaches the lens entrance, causing distraction. Figure 7b demonstrates the beveled baffle by cutting the symmetric baffle along a certain angle, thus the incident sunlight won’t affect imaging without a reflect surface.

According to the discussion in Section 3.1, considering the range of solar incidence angle δ during on-orbit task, the beveling angle relative to the lens optical axis should be less than δ. In this specific situation, the beveling angle is along the solar incidence direction, the angle between beveling plane and camera optical axis is chosen to be 50°, which is less than 52° with margin. Moreover, when angle β differs from 17° to 27°, the beveling plane rotates along the local orbit coordinate +*Z* axis by −22°, the longer baffle side faces to the sun most, thus barricades sunlight of the highest degree.

**Figure 8:**
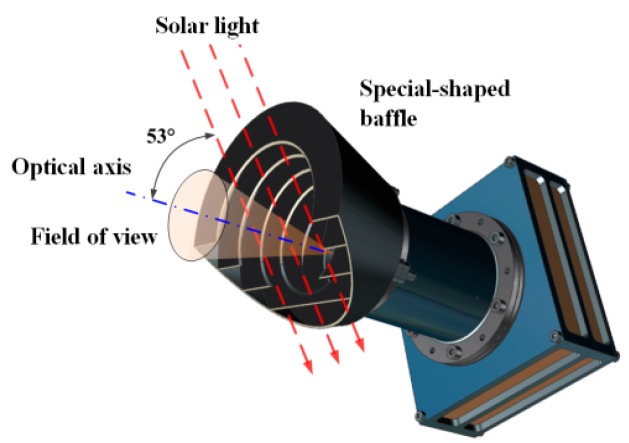
3D model of special-shaped baffle.

Camera baffle is used to restrain stray light comes directly from the sun as well as those from outside the field-of-view. The special-shaped baffle is of 58 mm in length and is painted black with an absorption rate as high as 96%. To minimize the environmental stray light from Path B in Figure 3, several vanes are designed inside the baffle to reduce the stray light energy entering the camera, as shown in Figure 8. However, it is worth mentioning that, although the special-shaped baffle is very useful to avoid solar light incident from some specified directions, this design may bring in some asymmetric response when the imaging scene is broadly illuminated, especially in laboratory radiometric calibration using integration sphere. This should be considered carefully in the radiometric calibration and validation process. 

## 4. Optimization of Lens Stray Light

As for stray light that already reaches the lens entrance, scattering on internal lens structure greatly affects the stray light transmission to sensor. The optimization of lens internal structure is thus necessary for restraining environmental stray light.

To effectively reduce the stray light, the corresponding elimination structure should be designed considering the stray light energy transmission. According to the theory of radiation energy conduction, light energy travels between two media surfaces is [36,37]:

$$d\emptyset_{c}=\frac{L_{s}\left(\emptyset_{0},\varphi_{0},\lambda_{0}\right)\cdot dA_{s}\cdot cos\theta_{s}\cdot dA_{c}\cdot cos\theta_{c}}{R^{2}}$$

where d$\emptyset_{c}$ is the flux on receiving surface, $L_{s}\left(\emptyset_{0},\varphi_{0},\lambda_{0}\right)$ the illuminance of source plane, $A_{s}$ and $A_{c}$ the area of both source and receiving surfaces. R is the length from center of source plane to that of receiving surface. The primary scattering is defined as the power transmission among surfaces with different Bidirectional Reflectance Distribution Function (BRDF) properties. BRDF is the ratio of the radiance $L_{s}\left(\emptyset_{0},\varphi_{0},\lambda_{0}\right)$ on output direction to the irradiance on input direction, it is used on describing the scattering feature of structure surface. The mathematical expression of primary scattering is:

(1) $$\emptyset_{c}=\pi \emptyset_{s}\left(BRDF\right)\left(GCF\right)$$

where $\emptyset_{c}$ is the power on receiving surface, $\emptyset_{s}$ is input power on the primary scattering surface. GCF is the Geometric Characteristic Factor, which is obtained as:

(2) $$\mathrm{GCF}=\frac{A_{c}cos\theta_{s}cos\theta_{c}}{\pi R^{2}}$$

According to Equation (2), the internal stray light can be restrained in three ways:

(1) Lower the input power by reducing the primary scattering surface area;
(2) Decrease the scattering by means of anodizing or painting extinction coating on mechanical structure;
(3) Set up vanes to reduce the GCF.

Stray light inside the lens consists of two parts, reflection from the lens side surfaces and the scattering of internal structures. We performed optimization on lens shape and reduced the scattering area on lens mount to restrain the non-target rays.

**Figure 9:**
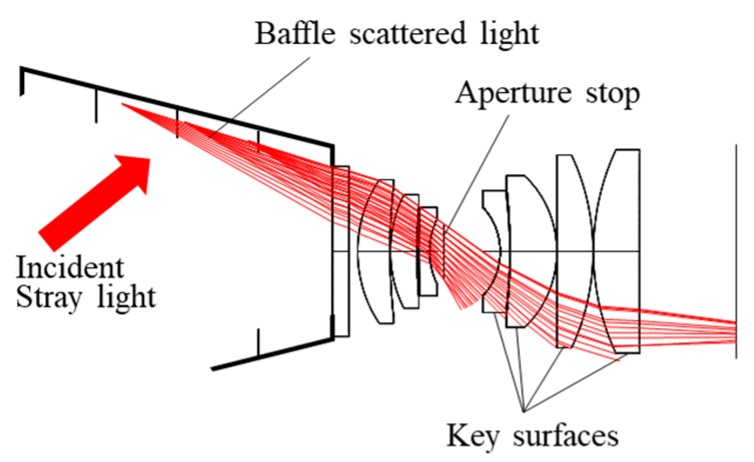
Key surfaces in optical system for stray light removing.

In detail, the prime stray light power transmit angle was obtained by ray tracing. Thus, it provided the stray incidence angle on each piece of lens, guiding us to optimize the structure respectively. The Luojia-1 camera with special-shaped baffle is shown in Figure 9. The ray tracing was performed regarding the interior baffle surface as the light source. The main stray light incidence direction and key surfaces of the optical system were found. It could be concluded that stray light went directly on the side surfaces of the lens pieces behind the aperture stop, producing scattered rays towards the sensor. The result revealed that these pieces were important in restraining stray light.

**Figure 10:**
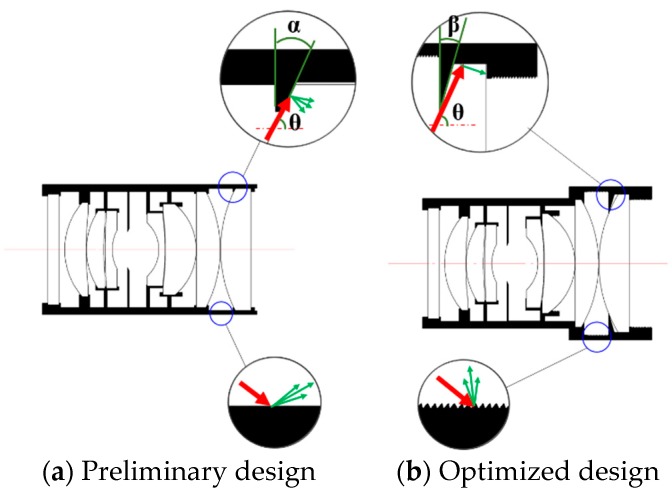
Optimization of lens internal structure.

According to total reflection theory, the side surface of the lens could reflect rays exceeding the critical angle, producing interior sources of stray light. It cannot be eliminated by painting. For this kind of stray light, the best way to restrain it is to expand the lens aperture and use a lens mount to cover up the excess part, cutting the path for stray light reaching the side face, as shown in lower circles in Figure 10. The last two pieces of the lens in Figure 10a are not optimized, while after optimization in Figure 10b the stray light is suppressed by an enlarged aperture and thread.

The lens mount edge that exposes in the light path is cut into a sharp edge to reduce the secondary scattering area. The angle of the sharp edge is designed as the ray incidence angle onto the stop to minimize the scattered energy, as shown in upper circles in Figure 10. When α+θ>90°, the incident stray light and the cut edge form an acute angle, scattering the stray light in the imaging path. While when β+θ<90°, the incidence angle is blunt, the stray light is then cut by the structure after passing the lens.

PST of the camera was analyzed in Tracepro software [38], the configurations of camera parts were as follows:

(1) Optical components: surfaces of the transmissive parts were set as 3-layer anti-reflective coating while the non-transmissive parts as black paint;
(2) Lens mount and clamping rings: Black paint.

**Figure 11:**
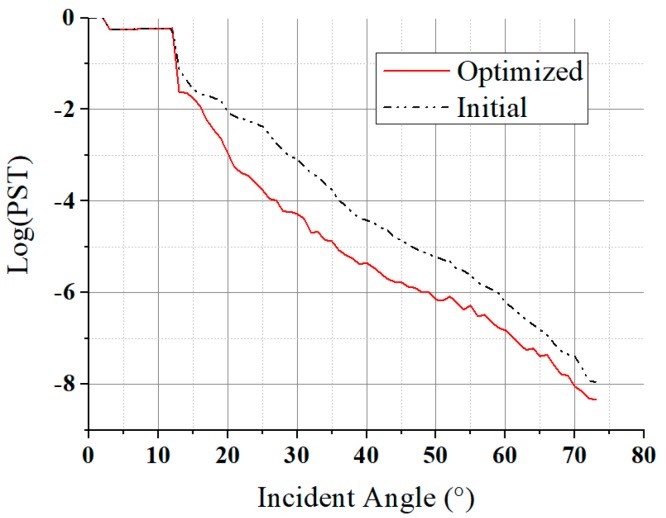
Evaluation of lens stray light after optimization.

The analysis results are shown in Figure 11. The optimization decreased the PST by an order in 20°–60° range, proving the structure optimization was efficient. 

## 5. PST Analysis Based on Whole-Satellite Environment

**Figure 12:**
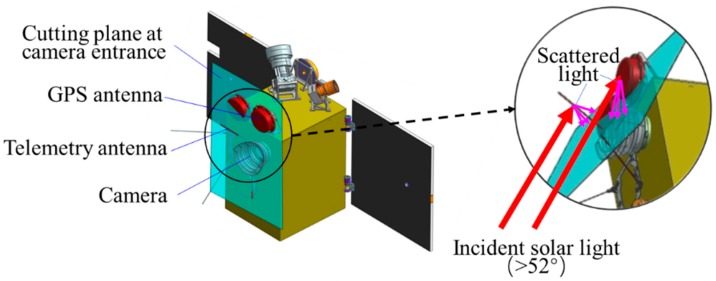
Camera environment affects stray light.

The Luojia-1 satellite is a microsatellite of small volume. The satellite’s earth facing surface is limited, and the TT&C and GPS antennas are close to the camera entrance. Hence, the solar and atmosphere radiation may be scattered by these surrounding parts into the camera, as shown in Figure 12. In the enlarged view, a transparent dummy surface parallel to the camera entrance is used to analyze visibility of onboard components. It showed that some parts of the GPS and TT&C antennas can be seen by the camera, providing paths for stray light to enter the camera. Therefore, it is necessary to analyze the stray light transmission with the whole-satellite set-up, which will give a more accurate evaluation on the camera’s real ability to eliminate stray light.

To improve analysis accuracy, the whole-satellite model was considered, the configurations of the model were as follows:

(1) Baffle: Black paint;
(2) Mounting plate, GPS and TT&C antennas, and data transmission antenna: Mirror reflectivity was 0.2, diffuse reflectivity was 0.4 and absorptivity was 0.4.

**Figure 13:**
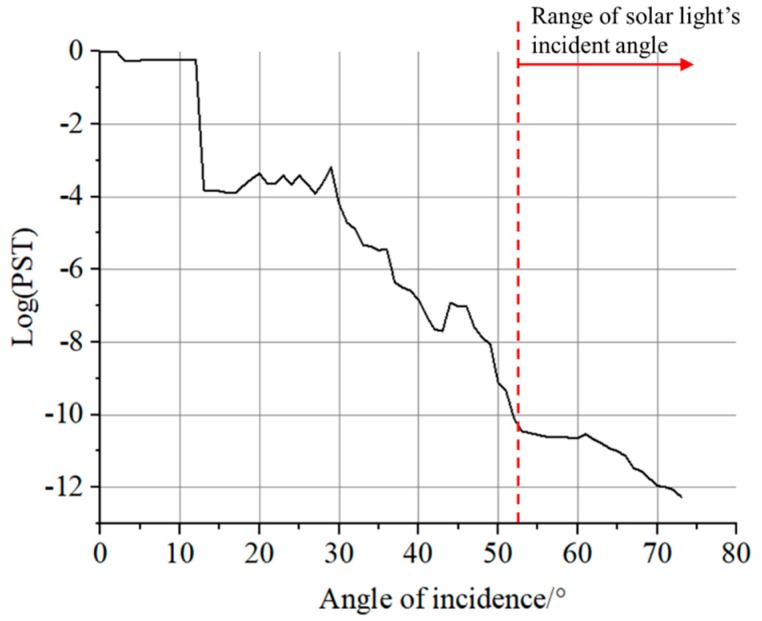
PST curve considering whole-satellite model.

The result of the PST curve under the whole-satellite set-up is shown in Figure 13. Unlike normal PST curves that are decreasing monotonically, this curve consists of some local uphill parts, which are caused by scattering on environmental satellite components near the camera, and multiple scattering of the baffle leaves. The PST within the solar incident range is less than order of 10^−10^, which is acceptable for our mission. It can be easily found that, if there was no special-shaped baffle in the simulation model, the system’s PST would be 10^−6^–10^−7^, as shown in Figure 11. When the special-shaped baffle was involved, the PST would be less than 10^−10^, as shown in Figure 13. Therefore, although there are other satellite components effecting, we can deduce that the proposed special-shaped baffle can at least decrease the stray light by three or four orders of magnitudes. 

The influence of moonlight can also be estimated using Figure 11 and Figure 13, because the moonlight also affects image quality during nighttime remote sensing imaging, especially the full moon. There are two ways of moonlight affecting nighttime images. One way is to incident into the camera as stray lights, the other way is illuminating the ground scene and making the image’s gray scale abnormal. For the first case, because of the 3-axis earth-oriented attitude used, the angle between the moon and the camera’s optical axis will be at least 67° (the angle from optical axis to the earth limb), the PST in Figure 11 for 67° is less than 10^−7^ (equivalently, there is no baffle, when moonlight incident from the baffle’s short edge). As we know, the radiance of the moonlight is about one over forty thousand of sunlight. Using the mathematical relationship deduced in Section 2, the maximum response of the moonlight is equal to ground illumination of 7.6 × 10^−4^ lux, which is much smaller than the system’s detection threshold and can be ignored. The second case widely exists for all nighttime working cameras. The ground illumination of the full moon is only 0.2 lux. Compared with the typical illumination of the ground with artificial lights (from tens to thousands lux), it is still small, but will indeed bring instability to the camera’s radiometric performance, which should be further calibrated and processed. The averaging of time sequential images is one of the solutions to remove the influences of moonlight on quantitatively detecting of artificial lights [25]. In this paper, we focus on the analysis and reduction of solar stray lights, because it is much more powerful than the other stray light sources. If the reduction of solar stray light cannot satisfy the requirement, the night time imaging will be totally sabotaged.

## 6. In-Orbit Imaging Test Results

**Figure 14:**
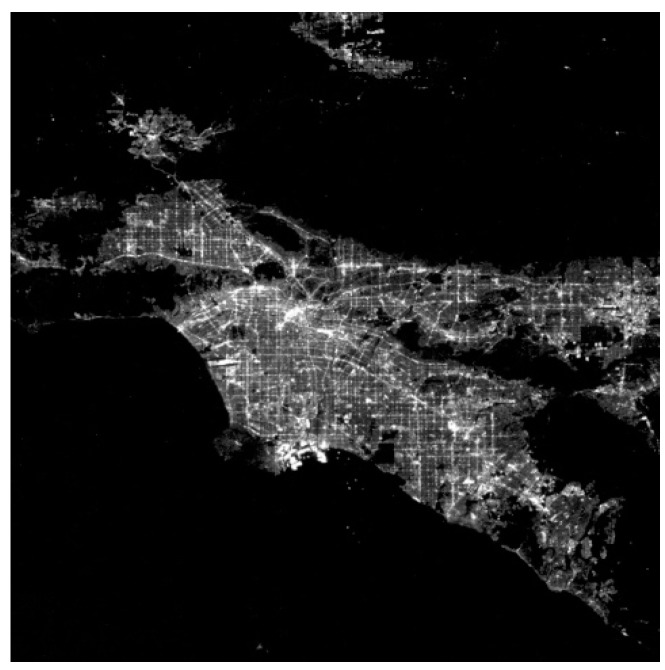
Nighttime image of Los Angeles (34° N), acquired with satellite in earth shadow area.

Figure 14 shows a typical nighttime image taken by Luojia-1 satellite. This is the first image acquired on the launch day, with the satellite in shadow area, free of solar stray light. The distribution of artificial lights and the structure of the city’s main roads could be well revealed. 

**Figure 15:**
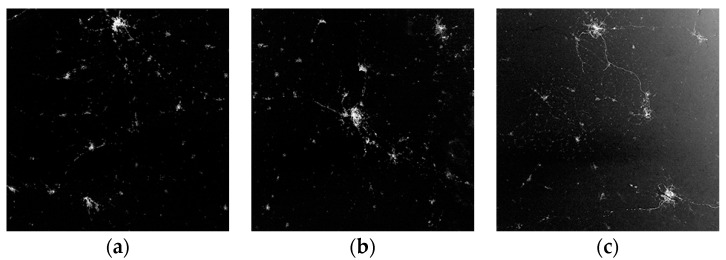
Different frames of nighttime image of the Moscow area (55° N), acquired when satellite was illuminated by sun on 21 June 2018. (**a**) Solar incident angel is 57°; (**b**) solar incident angel is 52°; (**c**) solar incident angel to optical is 47°.

In order to verify the solar stray light reduction performance of the camera, a push-broom imaging of high latitude area was carried out on 21 June 2018 and the images are shown in Figure 15. During this imaging of the Moscow area, the satellite was always illuminated by the sun. With an attitude maneuver, the angle between the optical axis and solar vector was decreasing during the whole progress. As is stated above, stray light will increase the gray scale of the image when it is larger than the camera’s detection threshold. Therefore, using the average DN (Digital Number) value of the image’s lightless area, we can evaluate the relative stray light response. The DN value for Figure 15a was only 8.6 (12-bit quantization) when its solar incident angle was 57°. This gray scale was similar with the lightless sea area in Figure 14, hence it can be considered that the solar stray light cannot be detected in Figure 15a. When the solar incident angle was deceased to 52°, as shown in Figure 15b, the DN value of lightless area was a little increased to 16.1, but there was still no obvious brightness increasing in the image. However, the image’s brightness was changed a lot when the angle was 47°, as shown in Figure 15c, whose DN value quickly reached to 105.3 in the middle and 277.6 in the margin, showing serious effect of solar stray light. These in-orbit test images showed that our design of the special-shaped baffle has an obvious effect on the solar stray light reduction, and its performance was preliminarily proved as predicted.

**Figure 16:**
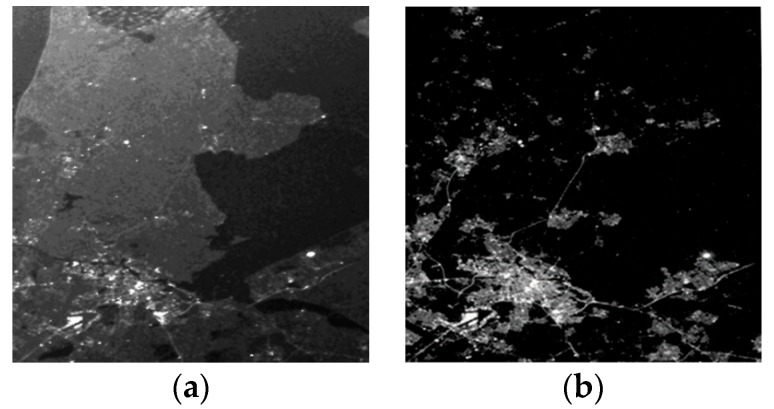
Images of Amsterdam (52° N), in different seasons. (**a**) Acquired on 29 June 2018; (**b**) acquired on 7 September 2018.

It is worth noting that, there is another phenomenon for nighttime imagers, which seems like the stray light influence, but it is totally different. It is caused by the practical illumination situation variations of the observed scene, so it can hardly be reduced by any hardware solution. An example is shown in Figure 16. These two images demonstrate the same area near Amsterdam in different seasons. When Figure 16a was acquired on 29 June 2018, the solar elevation angle for this area is nearly zero, hence the land and clouds were illuminated by sun and would reflect some sunlight towards the camera. Compared with Figure 16b, which was acquired on 7 September 2018, the terrestrial boundary on Figure 16a was much clearer. Obviously, although for Figure 16a with the satellite illuminated by sun, increase of the grayscale number on the image was not simply caused by solar stray light effects on the camera. The image just recorded the real scene as any camera can see at that time. This is quite different from the example given in Figure 15. 

As Figure 16a was acquired when sun illuminated on satellite, it was also evidence for the camera’s good stray light reduction performance, because the artificial lights can also be detected on the image with no evident of stray light pollution. However, for end users who want to quantificationally extract artificial light radiations from nighttime images, the gray level variations on images of different seasons will also bring serious problems. This kind of problem can hardly be avoided and makes precise measurements of artificial lights in the polar region very difficult in a polar day situation. It requires a lot of calibration, data cleaning, and processing. This topic was also mentioned in previous data research articles [27,39,40,41].

## 7. Conclusions

For high sensitivity nighttime cameras, solar stray light greatly affects the imaging process. Thus, it requires a whole system consideration of stray light sources, transmission paths, and environmental conditions. This article is based on the practical requirements of the Luojia-1 satellite and orbit analysis, a special-shaped baffle design, and the optimization of internal camera structure. The method of evaluating solar stray light based on the whole-satellite model are proposed, realizing the elimination of affection from solar stray light on sensing low illumination targets. The results of in-orbit tests prove the validity of the proposed solutions in this article. It guaranteed the successful nighttime image acquisition on specific areas by the Luojia-1 satellite.
